# Assessment of Acute Obstetrical Needs and the Potential Utility of Point-Of-Care Ultrasound in the North East Region of Haiti: A Cross-Sectional Study

**DOI:** 10.5334/aogh.2597

**Published:** 2020-07-03

**Authors:** Danica J. Gomes, Benjamin Kaufman, Adam R. Aluisio, Scott Kendall, Vladimir Thomas, Christina Bloem

**Affiliations:** 1 Department of Emergency Medicine, Division of International Emergency Medicine, SUNY Downstate Medical Center, Brooklyn, NY, US; 2 Department of Emergency Medicine, Division of Global Emergency Medicine, Columbia University, New York, NY, US; 3 Department of Emergency Medicine, Division of International Emergency Medicine, Alpert Medical School of Brown University, Providence, RI, US

## Abstract

**Background:**

Point-of-care ultrasound (POCUS) implemented through task shifting to nontraditional users has potential as a diagnostic adjuvant to enhance acute obstetrical care in resource-constrained environments with limited access to physician providers.

**Objective:**

This study evaluated acute obstetrical needs and the potential role for POCUS programming in the North East region of Haiti.

**Methods:**

Data was collected on all women presenting to the obstetrical departments of two Ministry of Public Health and Population (MSPP)-affiliated public hospitals in the North East region of Haiti: Fort Liberté Hospital and Centre Medicosocial de Ouanaminthe. Data was obtained via retrospective review of hospital records from January 1 through March 31, 2016. Trained personnel gathered data on demographics, obstetrical history, diagnoses, clinical care and outcomes using a standardized tool. Diagnoses *a priori*, defined as those diagnoses whose detection could be assisted with POCUS, included multi-gestations, non-vertex presentation, cephalopelvic disproportion, placental abruption, placenta previa, spontaneous abortions, retained products and ectopic pregnancy.

**Results:**

Data were collected from 589 patients during the study period. Median maternal age was 26 years and median gestational age was 38 weeks. The most common reason for seeking care was pelvic pain (85.2%). Sixty-seven (11.5%) women were transferred to other facilities for higher-level care. Among cases not transferred, post-partum hemorrhage, infant mortality and maternal mortality occurred in 2.4%, 3.0% and 0.6% of cases, respectively. There were 69 cases with diagnoses that could have benefited from POCUS use. Between sites, significantly more cases had the potential for improved diagnostics with POCUS at Fort Liberté Hospital (19.8%) than Centre Medicosocial de Ouanaminthe (8.2%) (p < 0.001).

**Conclusion:**

Acute obstetrical care is common and POCUS has the potential to impact the care of obstetrical patients in the North East region of Haiti. Future programs evaluating the feasibility of task shifting and the sustainable impacts of acute obstetric POCUS in Haiti will be important.

## Introduction

Haiti, one of the poorest and most underdeveloped countries in the Western Hemisphere (ranking 163^rd^ on the Human Development Index, far lower than any other country in the Western Hemisphere), often lacks the necessary infrastructure and medical resources to provide adequate and lifesaving patient care [1]. This becomes even more pronounced specifically in pregnancy and maternal child health outcomes. Maternal mortality was estimated to be 360 per 100,000 live births in 2015 compared to 92 per 100,000 live births in the Dominican Republic, a country located on the same island as Haiti [2]. The lifetime risk of maternal death is estimated at 1 in 80 [3]. The national under-5 mortality is 69 per 1000 live births, more than double that in the Dominican Republic (31 per 1000 live births) [4]. The percentage of under-5 deaths that occur in the neonatal period (0–28 days of life) is estimated at 34% [5,6]. These numbers are substantially worse than many other low- and middle- income countries (LMICs), particularly in the Western Hemisphere.

Ultrasonography is a diagnostic modality that is clinically useful in the care of pregnant women. It helps improve maternal mortality and outcomes as well as neonatal mortality and outcomes by allowing for early diagnosis and recognition of concerning features of pregnancies that may result in life-threatening sequelae. Several studies have shown the utility of ultrasound imaging in under-resourced settings, albeit not specifically in Haiti. A study done in Malawi found that 69% of ultrasound scans provided clinical utility by confirming diagnosis, ruling out diagnosis or resulting in a specific course of action [7]. Likewise, in another study from Cameroon, ultrasonography proved useful in 60.7–67.8% of cases [8]. Research carried out in Ghana showed that 81% of ultrasound studies added to clinical diagnosis and 40% influenced care [9]. These studies demonstrate the benefits of ultrasound in underserved and resource poor settings.

There is less data, however, for the utility of point-of-care ultrasound (POCUS), specifically, in LMIC settings. Additionally, POCUS is a novel application in Haiti as most ultrasound studies are performed by licensed radiologists, rarely used in acute settings, and are often challenging to obtain due to cost and low numbers of clinician providers. To date, most data regarding POCUS in Haiti are from the peri-earthquake period [10,11,12]. Currently, at Fort Liberté Hospital and Centre Medicosocial de Ouanaminthe, clinical obstetrical care is based mainly on history and physical examination with little use of adjuvant imaging.

POCUS can provide more accurate insight into pregnancy and fetal progression, allowing for earlier emergency and life-saving interventions if indicated, thus positively impacting maternal and neonatal outcomes and mortality. This cross-sectional observational study investigated the potential impact of POCUS with regards to acute obstetric care at two large public hospitals in the North East region of Haiti.

## Study Design and Methods

### Ethics Statement

This study was approved by the Institutional Review Board (IRB) of the State University of New York Downstate Medical Center and the Ministry of Public Health and Population (MSPP) from the North East region of Haiti. IRB reference number: 842211.

### Study Design, Participants, and Setting

This retrospective observational study took place at two MSPP-affiliated public referral hospital centers in the North East district of Haiti: Centre Medicosocial de Ouanaminthe and Fort Liberté hospital. All women known or suspected to be pregnant presenting to the acute obstetrical care area were eligible for inclusion. Children, defined as patients under the age of 18, and legal prisoners were excluded from this research study. Data from medical records were collected from January 1 to March 31, 2016. Research personnel collaborated with both study hospitals to obtain data on patient characteristics, management, and outcomes, prior to the implementation of POCUS.

According to prior data, the two study hospitals are responsible for the majority of vaginal deliveries and cesarean sections in the region and neither facility has consistent access to ultrasound services [12,13]. The acute care obstetric areas of both hospitals offer 24-hour nursing and physician care. At the time of data collection, neither hospital used POCUS to guide management.

### Procedures and Data Collection

A detailed data collection tool was used by personnel trained in study protocols. Data was obtained by reviewing hospital records and hospital registries for each patient who fulfilled the inclusion criteria from January 1 to March 31, 2016. The MSPP requires that every health care facility keep detailed registries recording specific data points. These data points include: date of care, name, date of birth, age, gestation and parity, blood pressure, diagnosis at admission, type of delivery, facilitative instruments such as forceps or suction device, complications, live or still birth, sex of the neonate, final outcome for neonate (discharged, deceased, transferred), and final outcome for mother (discharged, deceased, transferred). The variables included in the data collection tools used during this study include: age, reason for seeking care, suspected diagnosis and disposition of mother (vaginal delivery, cesarean section, admission to ward, discharged home, other) and, if available, confirmed diagnosis and complications as well as overall outcome (live birth, still birth, bleeding complications, maternal death). Possible diagnoses included multiple gestation, breech, transverse or cephalic presentation, placental abruption, placenta previa, spontaneous abortions, retained products and ectopic pregnancy as well as “other” where the health care provider can write an option not available on the data collection form.

### Statistical Methods

Data analysis was performed using STATA version 15.0 (StataCorp; College Station, USA). Categorical variables were explored using frequencies with percentages. Continuous variables were analyzed using medians with interquartile ranges (IQR). Descriptive analyses were undertaken for the overall study population and for subpopulations of patients stratified by hospital site. Inferential analysis compared variables based on hospital site. For categorical variables, differences were assessed using Pearson *X*^2^ tests. For continuous variables, frequency distributions were examined graphically and either independent sample *t*-tests or Mann-Whitney U tests were used for normally and non-normally distributed samples respectively. In all analyses, significance levels were set using Bonferroni correction to account for multiple testing [14].

To assess the potential impacts of POCUS in the study population, univariate and multivariate logistic regression models were used yielding odds ratios (OR) and adjusted odds rations (aOR) with associated 95% confidence intervals (CI). In regression models, two outcomes of interest were used: first, cases which required transfer for further treatment and evaluation, and second, the occurrence of delivery complications (which included: post-partum hemorrhage, perinatal death, and/or maternal mortality). The predictor variable was coded as binary as cases that could be assisted by the use of POCUS and in which clinical utility of POCUS would likely have existed or those that would not have been assisted by POCUS, and the likelihood of the outcomes of interest were calculated. The diagnoses of multiple gestation, breech presentation, transverse presentation, cephalopelvic disproportion, ectopic pregnancy, placental abruption, placenta previa, abortion and retained products were defined *a priori* as those in which POCUS could have impact on clinical outcomes. Regression models were run using the overall study population and for subpopulations stratified by hospital site. Missing data were addressed with listwise deletion in the regression analyses. As both maternal age and gestational age are known to impact health resource utilization and outcomes of mothers and infants, multivariate analyses were adjusted for these variables [15,16,17,18].

## Results

**Figure 1:**
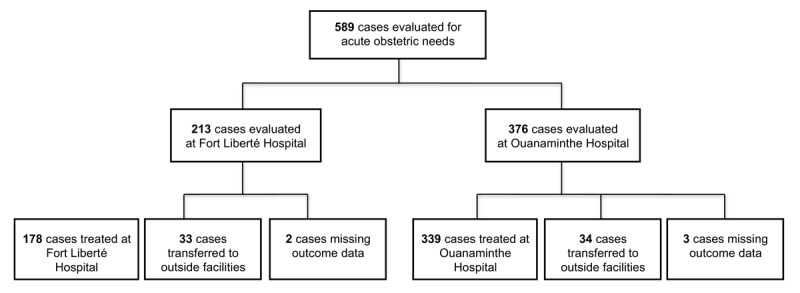
Study Population.

**Figure 2:**
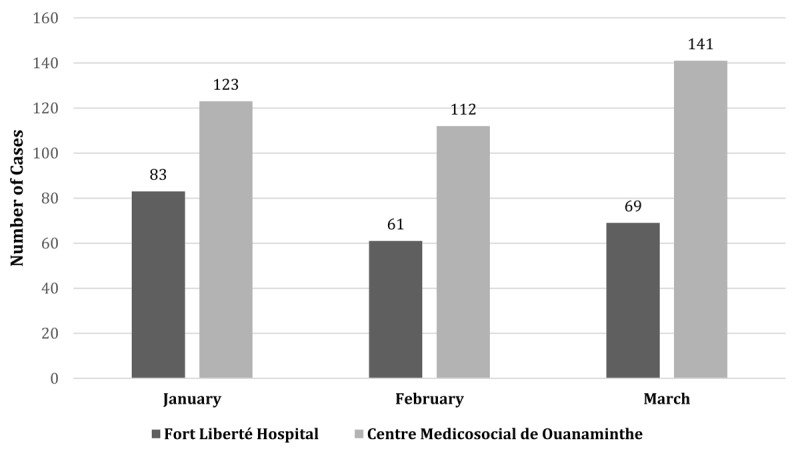
Frequency of cases presenting for care per month by hospital site.

Data were collected from the medical records of 589 patients who presented to the two study hospitals for acute obstetrical complaints. Five hundred seventeen (88.5%) cases were treated at these hospitals and 67 (11.5%) were transferred to outside facilities. At Fort-Liberté hospital, 213 cases were evaluated: 178 (84.4%) were treated in the hospital, 33 (15.6%) were transferred and 2 case charts had missing data and thus treatment status was unknown. At Centre Medicosocial de Ouanaminthe, 376 cases were evaluated in the hospital: 339 (90.9%) were treated on-site, 34 (9.1%) cases were transferred to outside facilities and 3 cases had missing treatment data (Figure 1). A higher frequency of cases were cared for at the Centre Medicosocial de Ouanaminthe as compared to Fort Liberté hospital over each month of evaluation (Figure 2).

The median age of patients studied was 26 years of age (IQR 22, 31 years). Among cases studied, the median gestational age was 38 weeks. A reason for presentation was identified for 487 (82.6%) cases. The most common presenting complaint was pelvic pain (85.2 %). The second most common reason for presentation was vaginal bleeding (3.5%). Of cases with an identified reason for presentation, 196 (40.2%) had two reasons. Prior to treatment, 554 (94.1%) cases had a suspected diagnosis. Among these, 69 (12.5%) of these diagnoses would have been aided by use of ultrasound (Table 1). The most common final disposition was vaginal delivery (80.8%), followed by transfer to another facility for further diagnostic studies and care. The prevalence of cesarean section in this cohort was 3.9%. The most common outcome was live birth at 94.0%, while the most common complication was perinatal mortality in 3.0% of cases, followed by post-partum hemorrhage in 2.4% of cases.

**Table 1:** Overall Cohort Characteristics.

Characteristics	n (%)/median (IQR)

Maternal age in years	26 (22, 31)
Gravidity	2 (1, 3)
Parity	1 (0, 2)
Gestational weeks	38 (36, 38)
Reason for care*	
pelvic pain	415 (85.2%)
vaginal bleeding	17 (3.5%)
vaginal discharge	10 (2.1%)
evaluation for pre-eclampsia	5 (1.0%)
evaluation of hypertension	12 (2.5%)
Suspect abnormal pre-treatment diagnosis	
multi-gestation	5 (0.9%)
breech presentation	8 (1.4%)
transverse presentation	3 (0.5%)
cephalopelvic disproportion	19 (3.4%)
ectopic pregnancy	0 (0.0%)
placental abruption	1 (0.2%)
placenta previa	3 (0.5%)
Abortion	28 (5.1%)
retained products	2 (0.4%)
Patient disposition	
birthing room for vaginal delivery	472 (80.8%)
operating room for C section	23 (3.9%)
admitted to ward for observation	12 (2.1%)
discharged for outpatient follow up	10 (1.7%)
transferred for further care	67 (11.5%)
Care outcome^#^	
live birth	469 (94.0%)
post-partum hemorrhage	12 (2.4%)
perinatal infant mortality	15 (3.0%)
maternal mortality	3 (0.6)

* Reason for obstetrical evaluation if not or in addition to concern for normal labor. ^#^ Provides data only for patients not transferred for further care.

Stratifying the data by hospital site, it was found that at Fort Liberté hospital, a reason for presentation was identified for 201 (94.3%) cases, and at Centre Medicosocial de Ouanaminthe for 286 (76.0%) cases (Table 2). Significantly more cases would have been aided by POCUS at Fort Liberté hospital (40 of 202 cases, or 19.8%) than at Centre Medicosocial de Ouanaminthe (29 of 352 cases, or 8.2%) (P < 0.001). In addition, cesarean sections were performed in 10.9% of cases at Fort Liberté compared to 0.0% of cases at Centre Medicosocial de Ouanaminthe. At Fort Liberté Hospital, 15.6% of patients were transferred, compared to 9.1% at Centre Medicosocial de Ouanaminthe. Lastly, at Centre Medicosocial de Ouanaminthe, there were 3 (0.9%) maternal deaths compared to Fort Liberté Hospital where there were 0 (0.0%) maternal deaths.

**Table 2:** Characteristics by hospital site.

Characteristics	Fort Liberte Hospital (n = 213)	Centre Medicosocial de Ouanaminthe (n = 376)	p

	n (%)/median (IQR)	n (%)/median (IQR)	

Maternal age in years	27 (23, 32)	26 (22, 31)	0.12
Gravida	2 (1, 3)	2 (1, 3)	0.13
Para	1 (0, 2)	1 (0, 2)	0.02
Gestational weeks	36 (34, 38)	38 (38, 38)	<0.001
Reason for care*			
pelvic pain	172 (85.6%)	243 (85.0%)	0.85
vaginal bleeding	12 (6.0%)	5 (1.8%)	0.021
vaginal discharge	6 (3.0%)	4 (1.4%)	0.408
evaluation for pre-eclampsia/eclampsia	3 (1.5%)	2 (0.7%)	0.393
evaluation of hypertension	9 (4.5%)	3 (1.1%)	0.033
Suspected abnormal pre-treatment diagnosis			
multi-gestation	3 (1.5%)	2 (0.6%)	0.360
breech presentation	3 (1.5%)	5 (1.4%)	1.00
transverse presentation	2 (1.0%)	1 (0.3%)	0.302
cephalopelvic disproportion	13 (6.4%)	6 (1.7%)	0.003
ectopic pregnancy	0 (0.0%)	0 (0.0%)	–
placental abruption	1 (0.5%)	0 (0.0%)	0.365
placenta previa	0 (0.0%)	3 (0.9%)	0.557
Abortion	18 (8.9%)	10 (2.8%)	0.002
retained products	0 (0.0%)	2 (0.6%)	0.536
Patient disposition			
birthing room for vaginal delivery	150 (71.1%)	322 (86.3%)	<0.001
operating room for cesarean section	23 (10.9%)	0 (0.0%)	
admitted to ward for observation	5 (2.4%)	7 (1.9%)	
discharged for outpatient follow up	0 (0.0%)	10 (2.7%)	
transferred for further care	33 (15.6%)	34 (9.1%)	
Care outcome^#^			
live birth	156 (89.7%)	313 (96.3%)	0.002
post-partum hemorrhage	9 (5.2%)	3 (0.9%)	
perinatal infant mortality	9 (5.2%)	6 (1.8%)	
maternal mortality	0 (0.0%)	3 (0.9%)	

* Reason for obstetrical evaluation if not or in addition to concern for normal labor. ^#^ Provides data only for patients not transferred for further care.

In multivariate regression analyses for the overall study cohort, there was an association with significantly increased likelihood that POCUS would have utility in enhancing care among patients transferred (aOR = 128.8, 95% CI: 17.0–975.3; p < 0.001). Likewise, this significant association was demonstrated for the utility of POCUS among patients with abnormal diagnoses (aOR = 41.4, 95% CI: 6.0–283.6; p < 0.001). When stratified by hospital site, similar associations were found in data from each clinical setting (Table 3).

**Table 3:** Evaluation of Potential Clinical Utility of Ultrasound.

	Outcomes			

	Transferred		Delivery Complications^β^	

	**Overall Cohort**			

	OR (95% CI)	aOR^¶^ (95% CI)	OR (95% CI)	aOR^¶^ (95% CI)

Diagnosis assisted by ultrasound^ɛ^	14.2 (7.80–25.7)*	128.8 (17.0–975.3)*	90.5 (33.5–244–6)*	41.4 (6.0–283.6)*
	**Fort Liberté Hospital Cohort**			

	**OR (95% CI)**	**aOR^¶^ (95% CI)**	**OR (95% CI)**	**aOR^§^ (95% CI)**

Diagnosis assisted by ultrasound^ɛ^	7.2 (3.2–16.2)*	131.9 (4.12–4226.0)^#^	21.2 (6.7–67.5)	19.1 (6.0–61.5)*
	**Centre Medicosocial de Ouanaminthe Cohort**			

	**OR (95% CI)**	**aOR (95% CI)**	**OR (95% CI)**	**aOR^§^ (95% CI)**

Diagnosis assisted by ultrasound^ɛ^	28.0 (11.2–70.1)*	126.0 (99.6–1663.2)	1074.3 (103.3–11173.6)*	725.5 (55.5–9477.3)*

^ɛ^ includes multiple gestation, breech, transverse, cephalopelvic disproportion, ectopic pregnancy, placental abruption, placenta previa, abortion and retained products. ^β^ includes post-partum hemorrhage, perinatal death, and maternal mortality. ^¶^ multivariate model adjusted for maternal age and gestational weeks. ^§^ multivariate model adjusted for maternal age. * associate p value < 0.001. ^#^ associated p value < 0.01.

## Discussion

This study demonstrates several of the top complications facing pregnant women in the North East region of Haiti: vaginal bleeding, pre-eclampsia, cephalopelvic disproportion, breech and transverse presentation, spontaneous abortions and multiple gestation. As suggested by the types of disease burdens observed and the current analysis, there is likely a potential for POCUS to improve the evaluation and treatment of patients in this study setting through early identification of disease processes and associated earlier interventions.

With non-cephalic and multiple gestation pregnancies, ultrasonography allows for better assessment of fetal presentation and early intervention when indicated. Cephalopelvic disproportion is another diagnosis that can be aided by ultrasonography as an adjunct to physical exam, thus allowing for more rapid escalation of medical and/or surgical care. Additionally, ultrasonography is one of the first steps in the diagnostic work-up for vaginal bleeding during pregnancy as well as for spontaneous abortion; ultrasound can assist with rapid diagnosis of placental previa, placental abruption, retained products and multiple other life-threatening etiologies of vaginal bleeding in pregnancy. Although not all pregnancy complications would benefit from ultrasound, several of the diagnoses documented in this study could be impacted by use of POCUS; therefore, the introduction of this diagnostic modality has substantial potential to contribute to improving care delivery and patient outcomes in the North East region of Haiti [7,8,9]. Further study with attention to provider type and implementation in task shifting to non-physician clinical providers will be of crucial importance given the health workforce composition in Haiti.

The infant mortality rate in Haiti is significantly higher than that of most other countries in the Caribbean and mirrors many under-resourced African countries [19]. This stems from a diverse set of underlying factors. Notably, the World Health Organization (WHO) sets a “medically necessary” target cesarean section rate at 10–15%. In this study in the North East region of Haiti, only 3.9% of patients received cesarean sections. POCUS may have a role in early identification of pathologies necessitating cesarean section within this population, thus leading to improved outcomes and decreased complications. Combining ultrasound as a clinical adjunct with improvements in prenatal, obstetric and postnatal care may improve outcomes in Haiti. Further prospective studies of the implementation of this key modality are needed.

In addition to the aforementioned reasons, POCUS may also be a beneficial diagnostic adjunct to acute care in Haiti as it allows for rapid imaging and identification of complications. Rapid diagnostic capacity would be particularly valuable in this population where health seeking behaviors often result in a lack of prenatal care and overall late presentation when illness occurs. Haiti has significant resource limitations and its population has a high poverty index [1]. These characteristics result in late presentations and an inability to access care in a time-sensitive manner. In addition, patients often lack the ability to pay for higher level examinations, (i.e. official prenatal ultrasound studies), that would provide healthcare workers with the information needed to develop the safest and most appropriate care plans. Therefore, maternity patients in Haiti have a higher incidence of life-threatening presentations requiring immediate interventions as well as a higher incidence of undiagnosed but easily identifiable pregnancy complications. POCUS could assist providers in overcoming some of these gaps by allowing for easier and more rapid identification of complications and diagnoses, thus facilitating the generation of medical care plans that would best serve the patient’s needs.

With regards to the differences between hospital sites in this study, Fort Liberte hospital had a much higher percentage of abnormal diagnoses that could have been aided by POCUS. The difference between these sites may be due to the fact that Fort Liberte Hospital is in a more rural location compared to Centre Medicosocial de Ouanaminthe. Centre Medicosocial de Ouanaminthe is located on the border between the Dominican Republic and Haiti and thus receives a large amount of vehicular traffic, which increases access to advanced medical and surgical care. In addition, official prenatal ultrasonography is offered more frequently at Centre Medicosocial de Ouanaminthe as opposed to Fort Liberte hospital allowing for abnormal findings to be identified earlier and be addressed more rapidly for patients presenting to Centre Medicosocial de Ouanaminthe.

In the current data, the multivariable regression models yielded large magnitudes of effect for the potential clinical utility of POCUS. Although the associations are statistically significant and likely valid, the point estimates are most likely larger than the true effect due to the underlying low numbers of events of interest and sample size, reflected by the wide confidence intervals. The importance in the data, however, lies not in the magnitude of the point estimates, but rather the association of POCUS with clinical utility. The findings in the present study demonstrate potential benefits of POCUS for pregnant patients, congruent with the current evidence in the literature which supports the validity of the results [20,21,22,23].

### Limitations

This study does have limitations that are intrinsic to the design. As a retrospective study, there is likely selection bias as only patients presenting for medical care could be included. Also, the data is limited to the details recorded by the health care worker during the consultation which was performed without knowledge of the specific question being analyzed. As a result, some data points are missing. Additionally, due to the general record keeping system in the study hospitals, it is possible that some patient visits were not documented or documentation was misplaced. Missing records should be minimal, however, as the MSPP requires strict documentation in a separate national registry of all births and complications surrounding each birth, a system that has been in place for many years. Although the multivariable analyses controlled for factors associated with the outcomes of interest, unmeasured confounders such as disease specific characteristics and variations in treatments may have influenced the findings. To address this limitation, alternative statistical methods could be employed such as the use of E-Value analytical approaches to assess for the effect of measured confounding, however the most optimal approach would be the completion of a dedicated prospective study of the implementation of POCUS in the study setting [24].

## Conclusion

This study evaluated the potential impact of POCUS for the care of obstetrical patients in the North East region of Haiti. Based on the data collected from these two public referral hospitals in this region, the introduction of a POCUS program may have great potential to provide clinically meaningful information to improve health outcomes in this population. Lessons learned in implementation should continue to influence best practices in Haiti, and more broadly, in similar under-resourced settings.

## Additional File

The additional file for this article can be found as follows:

10.5334/aogh.2597.s1 Appendix A. Data Collection Tool.

## Abbreviations

POCUS – point of care ultrasound

LMIC – low- and middle-income country

MSPP – Ministère de la Santé Publique et de la Population

IQR – interquartile range

OR – odds ratio

aOR – adjusted odds ratio

CI – confidence interval
