# Supplementary material for: Assessment of Acute Obstetrical Needs and the Potential Utility of Point-Of-Care Ultrasound in the North East Region of Haiti: A Cross-Sectional Study
Source: Ann Glob Health. 2020 Jul 3;86(1):72. doi: 10.5334/aogh.2597 (PMC7333557; doi:10.5334/aogh.2597)
Supplement: Appendix A. — Data Collection Tool. [file agh-86-1-2597-s1.pdf]

APPENDIX A: Data Collection Tool

1. ID number: __ _ __ __

2. Age (years): _ _ _

3. Pregnant (circle one):
   yes no unknown

4. LMP (day/month/year): _ _ / _ _ / 20 __ __

5. Gestation (number of prior Pregnancies): _ _

6. Number of deliveries:__ __

7. Reason seeking care (circle one):

Injury	Vaginal bleeding	Pelvic/abdominal pain	Vaginal discharge or fluid leakage	Delivery

Other: ______________________________

8. Suspected diagnosis – *to be completed during initial evaluation* (circle all that apply):

Multiple gestation	Breech	Transverse	Fetal demise	Ectopic pregnancy
Single gestation	Vertex	Placenta previa	Normal labor	Other:

9. Would an ultrasound study be performed, if available (circle one)? Yes No

10. Patient sent to (circle one):

Home	Operating theater/ cesarean section	Birthing room /vaginal delivery	Admitted to ward

Other: ______________________________

11. If the patients had a "cesarean section" or "vaginal delivery" what occurred (circle all that apply):

Live birth	Still birth	Single gestation birth	Multiple gestation birth
Breech presentation	Vertex presentation	Bleeding complications	Maternal death

Other: ______________________________
